# A Flexible Pressure Sensor Based on Magnetron Sputtered MoS_2_

**DOI:** 10.3390/s21041130

**Published:** 2021-02-05

**Authors:** Xing Pang, Qi Zhang, Yiwei Shao, Mingjie Liu, Dongliang Zhang, Yulong Zhao

**Affiliations:** State Key Laboratory for Manufacturing Systems Engineering, Xi’an Jiaotong University, Xi’an 710049, China; px2014@stu.xjtu.edu.cn (X.P.); imporeed@stu.xjtu.edu.cn (Y.S.); liumingjie@stu.xjtu.edu.cn (M.L.); zhangdl666@stu.xjtu.edu.cn (D.Z.); zhaoyulong@xjtu.edu.cn (Y.Z.)

**Keywords:** MoS_2_ sensor, magnetron sputtering, piezoresistive, flexible sensor

## Abstract

Although two-dimensional (2D) layered molybdenum disulfide (MoS_2_) has widespread electrical applications in catalysis, energy storage, and photodetection, there are few reports available regarding sputtered MoS_2_ for piezoresistive sensors. In this research, we found that the resistance of magnetron sputtered MoS_2_ on a flexible substrate changed significantly and regularly when pressure was applied. Scanning electron microscope (SEM) and atomic force microscope (AFM) images revealed an MoS_2_ micro-grain-like structure comprising nano-scale particles with grooves between the particles. Chemical characterization data confirmed the successful growth of amorphous MoS_2_ on a polydimethylsiloxane (PDMS) substrate. A micro-thickness film flexible sensor was designed and fabricated. In particular, the sensor with a 1.5 μm thick polydimethylsiloxane (PDMS) substrate exhibited the best resistance performance, displaying a maximum ΔR/R of 70.39 with a piezoresistive coefficient as high as 866.89 MPa^−1^ while the pressure was 0.46 MPa. A proposed flexible pressure sensor based on an MoS_2_ film was also successfully used as a wearable pressure sensor to measure plantar pressure and demonstrated good repeatability. The results showed that the thin film pressure sensor had good piezoresistive performance and high sensitivity.

## 1. Introduction

As an important component of a signal acquisition unit, in a signal conversation, sensors act in the role of responding to external incentives. In recent years, increasing signal acquisition and processing requirements under special circumstances have been demanded, and flexible sensors are playing an increasingly important role in electronic skin [1], intelligent wearables [2], medical care [3], industry electronics fields [4], etc. A flexible sensor has good flexibility and ductility and can bend freely; therefore, it is very convenient to detect the complex structure.

At present, carbon nanotubes (CNTs) [5,6,7] and graphene [8,9,10] are commonly used in flexible sensor sensitive film materials. They are used as excellent materials for flexible sensing sensitive films due to the advantages of high crystallinity, good electrical conductivity, and large specific surface area. CNTs and graphene must be transferred in the process of sensor production [11,12,13], which is difficult to achieve in mass production. There is an urgent need for developing new sensors with novel materials that not only have the function of flexible sensing but are also easy to prepare and can be mass-produced.

MoS_2_ is a novel functional material used as a transition metal disulfide (TMDs). MoS_2_ is a typical two-dimensional material of a stratiform semiconductor. Its structure is similar to the atomic layer of two-dimensional graphene, which consists of two layers of sulfur atoms on the outside and one layer of molybdenum in the middle [14,15,16,17,18,19,20,21]. MoS_2_ has the advantages of excellent electrical and optical properties, an unusual specific surface area, and a high direct bandgap up to 1.8 eV [22]; thus, it has great application potential in the field of micro-nano sensor technology.

In 2011, the Andras Kis research team of the Swiss Federal Institute of Technology produced the first batch of transistors with a single layer of 0.65 nm molybdenum disulfide (MoS_2_), which was expected to improve Moore’s Law [22,23]. Until 2014, the research of two-dimensional molybdenum disulfide as a piezoresistive material was gradually developed [24,25,26,27,28]. 

Considering that the atomic layer thickness of molybdenum disulfide has a high Young’s modulus and fracture strength, Andras Kis’s research team prepared 1–3 layers of mechanically peeled molybdenum disulfide into Nano-Electromechanical System (NEMS) strain sensors and found that large strains could adjust MoS_2_. The piezoresistance factor of the double-layer molybdenum disulfide is −224 ± 19 [24]. The Minhoon Park research team proposed an ultra-thin conformal tactile sensor based on MoS_2_, where MoS_2_ is the sensitive element, and graphene is used as the electrode for interconnection, showing good performance [26].

Two-dimensional MoS_2_ can be prepared using the micromechanical stripping method, lithium-ion intercalation stripping method, liquid-phase ultrasonic stripping method, hydrothermal method, and chemical vapor deposition [29,30,31,32,33]. Although all of these methods can obtain two-dimensional MoS_2_, it is difficult to obtain large-area single-layer MoS_2_ with the micromechanical stripping method, as the length is within several microns to tens of microns generally, and the yield is low [22]. The residual Li^+^ on the MoS_2_ surface affects the electrochemical performance to a certain extent through the lithium-ion intercalation stripping method, and the reaction period is long [34].

The liquid-phase ultrasonic stripping method is easy to operate but has a great influence on the quality of MoS_2_ obtained in the solvent selection and proportion control [35]. It is harsh to prepare MoS_2_ using chemical vapor deposition (CVD), and it needs to be prepared in a high-temperature environment [26]; however, the molybdenum disulfide piezoresistive pressure sensors described in the abovementioned documents are mostly prepared by these methods [22,23,24,25,26,27,28]. In comparison, the sputtering method is easy to control, the deposition rate is high, the film formation is dense and uniform, and it is easy to mass produce.

Here, an MoS_2_ film was prepared on the surface of a polydimethylsiloxane (PDMS) flexible substrate material through magnetron sputtering. The structure and morphology of the MoS_2_ film at a sputtering power of 350 W are discussed, and its piezoresistive performance was tested. It is shown that the devices have good piezoresistivity and high sensitivity to strain. MoS_2_ was grown directly on flexible PDMS films using the magnetron sputtering method, and the electrode was fabricated by screen printing. The sensor exhibited excellent sensitivity and repeatability. The practicability of the manufactural sensor was successfully evaluated in real samples through the human foot pressure test, thus holding tremendous potential as a simple and easy to achieve sensor in human health monitoring and rehabilitation medicine.

## 2. Materials and Methods

### 2.1. PDMS Materials

Polydimethylsiloxane (PDMS) is a kind of hydrophobic organic silicon material. It is one of the most promising materials for flexible sensors in the laboratory due to its excellent insulating transmittance and chemical stability [36,37]. Silicone elastomer kit (SYLGARD 184, DOW chemical company, Wiesbaden, Germany) is the material which we chose to compound the polydimethylsiloxane (PDMS). It consisted of a basic component and a curing agent. The basic component and curing agent were stirred at a mass ratio of 10:1. The mixture was placed in a vacuum environment of −0.1 MPa until there were no bubbles and left for later use. 

### 2.2. Preparation of Flexible Substrate

The flexible substrate was prepared using 4-in normal glass as the substrate. Before preparation, the glass was placed in acetone and ethanol for ultrasonic cleaning for 5 min. Then, the surface was rinsed with deionized water. To better realize the peeling of the flexible substrate and the glass, the glass surface was cleaned again using the Pink V6-G plasma cleaning equipment. The power was 300 W, the oxygen input was 150 mL/min, and the run time was 50 s. After that, the release agent was sprayed evenly on the glass surface and dried. 

**Figure 1:**
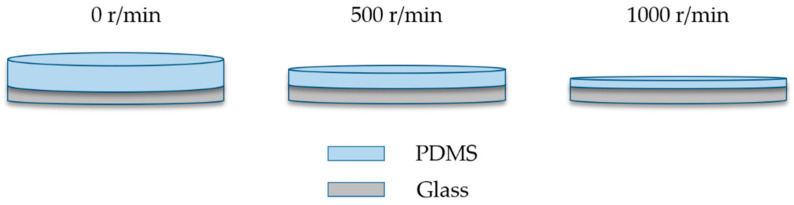
Diagram of the prepared polydimethylsiloxane (PDMS) substrates prepared with three different rotation speeds (0, 500, 100 r/min).

**Figure 2:**
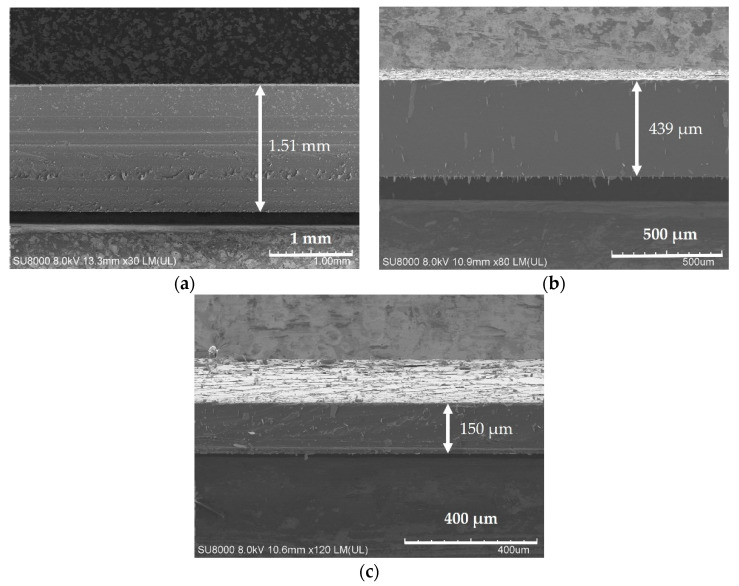
Scanning electron microscope (SEM) micrographs of the cross-sectional morphology of the PDMS substrates prepared with the three different spinning speeds. (**a**): The cross-sectional of PDMS thickness with 1.51 mm at the rotation speed of 0 r/min; (**b**): The cross-sectional of PDMS thickness with 439 μm at the rotation speed of 500 r/min; (**c**): The cross-sectional of PDMS thickness with 150 μm at the rotation speed of 1000 r/min.

Finally, the configured PDMS mixture was spread statically (the spinning speed at 0 r/min) and was spread at the spinning speeds of 500 r/min and 1000 r/min for 40 s on the prepared glass. As shown in Figure 1, three different thickness flexible substrates were produced. In Figure 2, we can see the scanning electron microscope (SEM) micrographs of the cross-sectional morphology of the different thickness PDMS samples. The thicknesses were approximately 1.51 mm, 439 μm, and 150 μm corresponding to the spinning speeds at 0, 500, and 1000 r/min, respectively. Three different thickness flexible substrates were, thus, prepared and provided the substrates for the preparation of the flexible sensor.

### 2.3. Growth of MoS_2_ on PDMS

**Figure 3:**
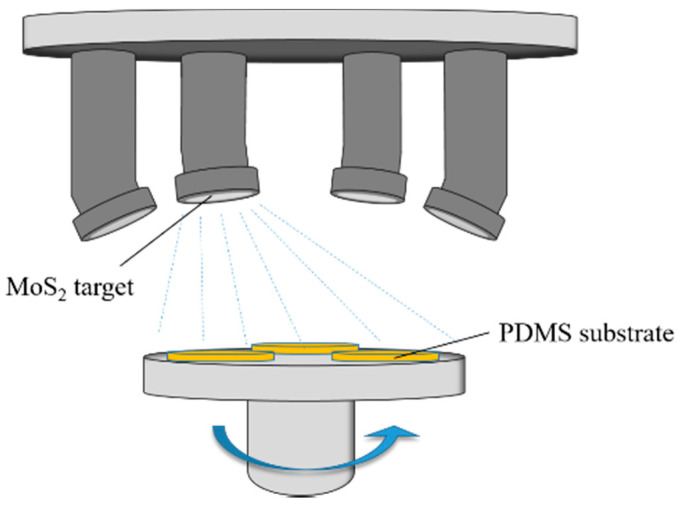
Schematic diagram of the molybdenum disulfide (MoS_2_) thin film sputtering deposition system.

The DISCOVERY635 Discovery Magnetron Sputtering system, produced by the American company DENTON, was used to deposit the MoS_2_ films in this experiment. As shown in Figure 3, four target guns can be installed on the top of the vacuum chamber, which can automatically switch to all four sputtering target guns to realize DC (Direct Current) sputtering. The target material for sputtering was an MoS_2_ target with a purity of 99.999%, and the target diameter was 101.6 mm. Before deposition, the prepared PDMS substrates were cleaned with Absolute Ethanol and deionized water, respectively.

After drying, the PDMS substrates were modified by an oxygen plasma cleaning machine to improve the adhesion between MoS_2_ film and the PDMS substrate [38,39]. Then, a cleaned PDMS substrate was placed on the sample platform in the vacuum chamber. When the vacuum in the room reached 2.6 × 10^−6^ Torr, high-purity Ar was injected into the chamber, and the gas flow was controlled to be 30 SCCM (Standard Cubic Centimeter per Minute). The working pressure in the room was adjusted to 5 m Torr. The MoS_2_ thin film was prepared with the sputtering power of 350 W at room temperature.

### 2.4. Characterization of MoS_2_ Film

An INNOVA atomic force microscope was used to analyze the surface roughness of the PDMS substrates and sputtered MoS_2_ thin films. The surface energy of the PDMS substrates was measured qualitatively through a JC2000 contact Angle analyzer produced by Shanghai Zhongchen Digital Technic Apparatus Co. Ltd. (Shanghai, China). A Field Emission Scanning Electron Microscope (Hitachi SU-8010, Tokyo, Japan) at an accelerating voltage of 7 KV was used to study the morphology of the thin film. The DXR™2xi Raman Imaging Microscope by ThermoFisher Scientific was used for Raman spectroscopy analysis. 

The laser wavelength and laser power were 532 nm and 0.7 mW, respectively. The phase analysis of the film sample was carried out using an X-ray Powder Diffraction (XRD, Bruker D8 Advance A25). The tube’s voltage and current were 40 KV and 40 mA, respectively. The target material used in the XRD test was a copper target with a wavelength of λ = 1.5418 nm. The goniometer was configured for 2θ operation at the test range between 10° and 80° (2θ = 10°–80°) with step size 0.02°. The grazing incidence mode was used in the test. The fixed incidence angle was 1°. An X-ray photoelectron spectrometer, Thermo Fisher ESCALAB Xi+, was used for the analysis of the composition and valence of the thin film.

### 2.5. Piezoresistive Performance Test of MoS_2_ Film

A screw mechanical pressure test bench was used to measure the piezoresistive properties of the MoS_2_ films, and the output force was read with a digital pressure gauge. The force was applied to the sensitive unit (MoS_2_ film) of the flexible pressure sensor through an insulating indenter. The size of the indenter was 1.0 × 1.0 cm^2^, which was larger than the size of the MoS_2_ film. The electrodes were made of conductive silver paste. The sample was pasted on the glass slide and then placed on the load table of the pressure test bench. The MoS_2_ film was connected to a multimeter to record the resistance synchronously when pressure was applied to it. Therefore, it provided a real-time output of the pressure and resistance. 

## 3. Results and Discussion

### 3.1. The Adhesion between MoS_2_ Film and PDMS Substrate 

**Figure 4:**
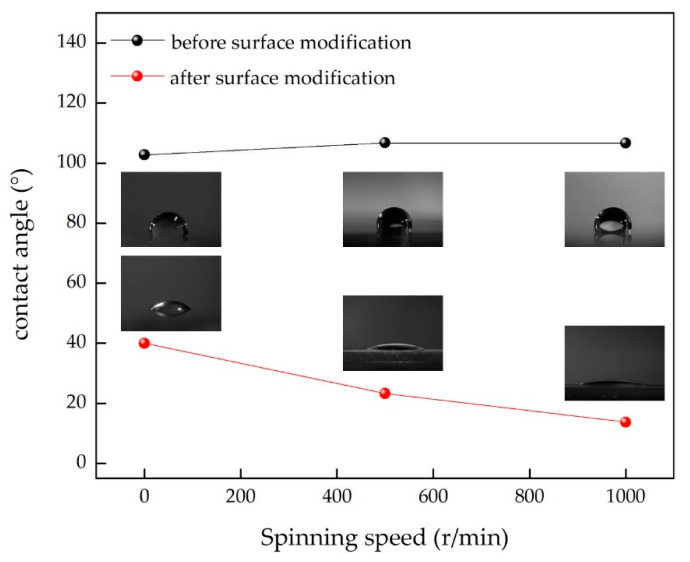
The surface contact angle of PDMS films at the three studied rotation speeds.

To improve the interface adhesion, the PDMS substrate surfaces were treated with oxygen plasma. The surface energy of the PDMS was measured by the contact angle. The contact angle is closely related to the surface wettability and surface energy [40,41,42]. Oxygen plasma treatment technology is a common means of surface modification of PDMS. The contact angle of a PDMS surface can be changed after oxygen plasma treatment, which can effectively improve the surface energy of PDMS [43,44]. Figure 4 shows the contact angles of PDMS films prepared at different spinning speeds before and after surface modification. 

We found that the contact angle was reduced significantly after oxygen plasma treatment. The surfaces of PDMS films treated without oxygen plasma were hydrophobic, and the contact angles were greater than 100°, while the surfaces of PDMS films treated by oxygen plasma were hydrophilic. We dropped 2.5 μL water on the modified PDMS, which spread out immediately on the surface of the PDMS substrates treated by oxygen plasma. The contact angles of the PDMS films prepared at the rotational speeds of 0, 500, and 1000 r/min were measured to be 40°, 23.3°, and 13.75°, respectively. Reducing the contact angle can increase the surface energy of PDMS effectively, which is beneficial to the deposition of an MoS_2_ thin film. 

Next, to further investigate the bonding strength between the PDMS substrate and the sputtered MoS_2_, a crosscut tape test was conducted. The PDMS substrate prepared at 0 r/min was chosen. The MoS_2_ film of 1 μm thick was sputtered at 350 W power on the oxygen plasma-treated and untreated PDMS substrates. Then, 100 grids with dimensions of 1 × 1 mm^2^ were drawn on the surface of the MoS_2_ film in different positions, and each line of the grid was deep to the bottom of the film. Second, a specified tape (Scotch 600, 3 M, St Paul, MN, USA) was firmly pasted on the films and then peeled rapidly and continuously. 

**Figure 5:**
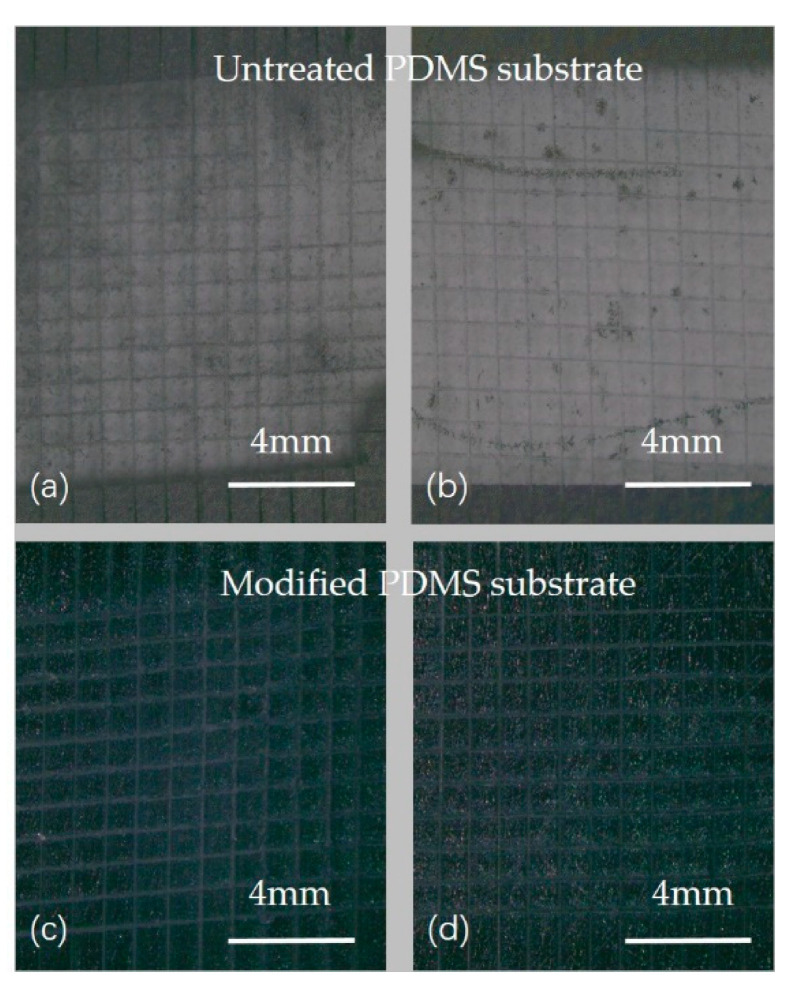
Photographs of the specimens after the crosscut tape test. (**a**,**b**): The MoS_2_ sputtered on PDMS substrate without oxygen plasma treatment was almost completely detached; (**c**,**d**): The MoS_2_ sputtered on oxygen plasma treated PDMS substrate was intact.

As shown in Figure 5, the sputtered MoS_2_ films on the untreated PDMS substrate were all peeled off. There was no peeling on the films sputtered on the modified PDMS substrate through oxygen plasma, and the edges of the grids were smooth as well. This indicates that the bonding strength between the modified PDMS substrate by oxygen plasma and the sputtered MoS_2_ film is high. Therefore, before sputtering MoS_2_ on the surface of PDMS, we can use the oxygen plasma equipment to modify the surface of PDMS, which can improve the bonding force of sputtered MoS_2_ and the surface of PDMS effectively. 

### 3.2. Characterization of PDMS and MoS_2_ Film

**Figure 6:**
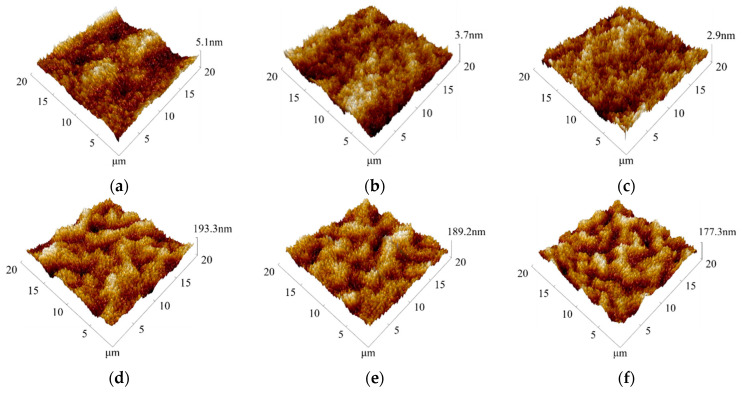
Atomic force microscope (AFM) surface morphology of the PDMS thin films and the MoS_2_ thin films deposited on PDMS at different rotation speeds. (**a**–**c**): AFM surface morphology of the PDMS thin films at rotational speeds of 0, 500, and 1000 r/min, respectively; (**d**–**f**): AFM surface morphology of the MoS_2_ thin films deposited on PDMS.

**Figure 7:**
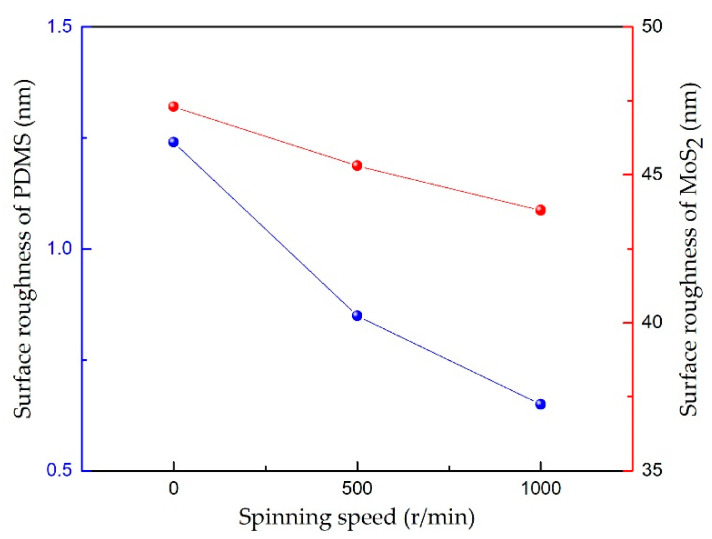
The surface roughness at different spinning speeds.

Figure 6a–c shows atomic force microscope (AFM) topographic images of the PDMS film on glasses spin-coated at different rotation speeds. The test range on samples for each film was 20 × 20 μm^2^. From Figure 6a–c, the surfaces of the PDMS films had the appearance of tiny peaks and valleys. The values of the surface roughness of the films were 1.24, 0.849, and 0.649 nm. This shows that the surface roughness of PDMS was smoother with the higher speed of the spin. Figure 6d–f shows AFM topographic images of the MoS_2_ deposited on the surface of PDMS thin films. It can be seen that MoS_2_ films covered the PDMS surface, which presented a crisscrossed appearance, like a mountain range, with dense nano-level grains. After MoS_2_ was deposited with about 1 μm thickness on the PDMS substrate at the power of 350 W, the surface roughness of the MoS_2_ films increased significantly. The values of the surface roughness were 47.3, 45.3, and 43.8 nm, which corresponded to the values of surface roughness of PDMS without sputtered, which were 1.24, 0.849, and 0.649 nm, respectively. As shown in Figure 7, both the PDMS film at 0 spinning speed and the MoS_2_ sputtered on its surface had the highest value of surface roughness. The lower the spinning speed, the larger the roughness of the PDMS surface, and the larger the roughness of the sputtered MoS_2_ film. The dense nano-level grains and larger roughness of the MoS_2_ film could increase the surface-to-volume ratio of the MoS_2_ film, and this could cause the strains and adsorption of the surrounding gas molecules on the surface [44], leading to a more significant piezoresistive effect.

**Figure 8:**
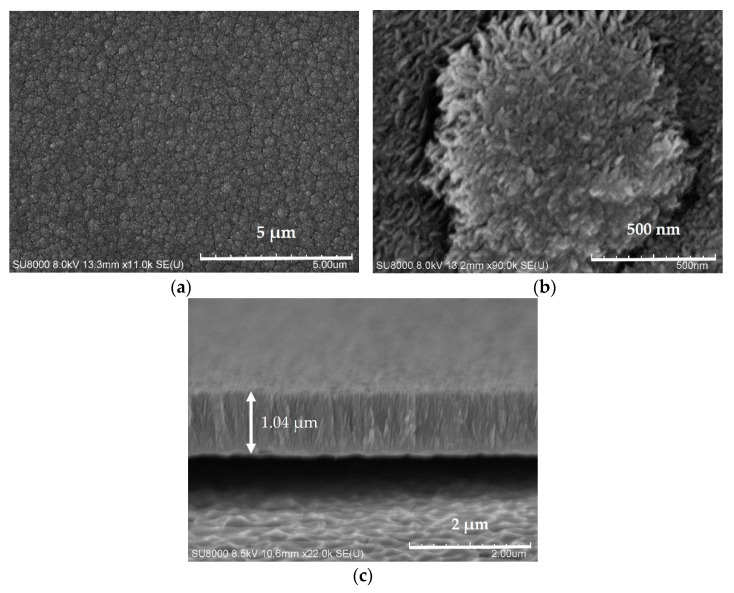
SEM micrographs of the surface and cross-sectional morphology of the MoS_2_ films deposited on PDMS substrates at the power of 350 W; (**a**,**b**) lower and higher magnifications, respectively; (**c**) the cross-sectional image of sputtered MoS_2_ film.

The surface morphology of sputtered grown MoS_2_ on the flexible PDMS substrate was characterized using SEM. Figure 8a shows the SEM image of MoS_2_ on PDMS substrates at room temperature using 350 W sputtering power. A mass of micro-grain-like morphology with the average sizes of 200–500 nm was observed. This was consistent with the observations that some researchers have made [45,46]. It was demonstrated, from the SEM image, that densely packed nano-scale particles formed an MoS_2_ thin film sputtered by magnetron sputtering, and there were grooves between the particles. In the magnetron sputtering process, Ar ions have higher kinetic energy under an electric field. 

When bombarding the target material, the escaped MoS_2_ molecules or molecular groups gained higher energy, and the energy reaching the substrate was higher. The migration ability on the substrates is vital; therefore, the film’s growth was relatively dense. The surface of the deposited film was bombarded by energy-carrying particles for a long time, leading to defects and resulting in the surface nucleation of the flat area and the pit morphology, as shown in Figure 8a [47,48]. In Figure 8b, there were one or more crystal pillars on a micro-grain, and the sputtered MoS_2_ had a crystal structure on a nano-scale. The cross-sectional image of sputtered MoS_2_ is shown in Figure 8c, and the thickness of the MoS_2_ film was approximately 1 μm. It was grown with a compact sharp tooth-like structure [49]. 

**Figure 9:**
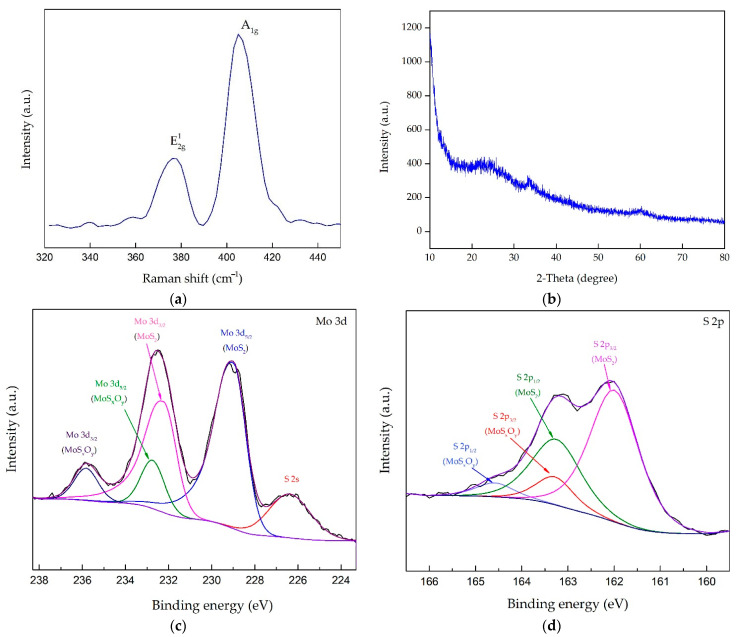
(**a**) Raman spectrum of MoS_2_; (**b**) X-ray powder diffraction (XRD) pattern of MoS_2_ grown on PDMS substrates; (**c**,**d**) Mo3d and S2p X-ray photoelectron spectroscopy (XPS) spectra of MoS_2_.

Detailed physical and chemical characterization were conducted using a Raman spectrometer, and XRD and X-ray photoelectron spectroscopy (XPS) were performed. The detection laser wavelength was 532 nm in the Raman spectrum test. Figure 9a displays the Raman pattern of the grown MoS_2_ on the flexible PDMS substrate, which sputtered a 1 μm thick MoS_2_ film at the sputtering power of 350 W. The characteristic peaks at 377.95 cm^−1^ and 405.23 cm^−1^ were accredited to the typical E12 g and A1 g peak vibrational modes of MoS_2_, respectively [50,51]. MoS_2_ has 12 vibrational modes of the crystal structure. 

The E12 g is an in-plane vibrational mode, and the A1 g is an out-of-plane vibrational mode, which are the two vibrational modes that can be easily observed. We can see from Figure 9a that the peak distance was 27.28 cm^−1^ longer than the single-layer MoS_2_ standard spectrum [52]. Due to this, the van der Waals force and coupling interaction between the layers increased gradually with the increasing sample thickness, leading to the shift of the A1 g and E12 g vibrational modes. The A1 g vibrational stiffened (blueshift), while the E12 g vibrational softened (redshift) [53], influencing that the energy difference enhanced gradually and the peak distance increased between the vibrational modes of A1 g and E12 g.

Figure 9b presents the XRD spectrum of the sputtered MoS_2_ on the flexible PDMS substrate. Two low-intensity diffraction peaks at 2θ = 33.82° and 59.93° were indexed to the (100) and (110) crystallographic plane of MoS_2_ [54,55]. The (100) crystallographic plane of MoS_2_ was attributed to the rhombohedral phase of MoS_2,_ and the (110) crystallographic plane was ascribed to the hexagonal phase of MoS_2_ [56,57]. The results showed that sputtered MoS_2_ grown on PDMS presented as mainly amorphous and grown with the (100) and (110) crystallographic plane, which could be due to the overlapping of crystallographic planes of both PDMS and MoS_2_. To further explore the chemical composition and the oxidation state of Mo in MoS_2_, XPS analysis was performed. The data extracted from the spectra of the films are shown in Table 1.

These include the binding energies, peak FWHMs, and atomic percentages determined by fitting XPS peaks to Voight functions. We found that the ratio of Mo and S was close to 1:2. Figure 9c shows the high-resolution XPS energy spectrum of the Mo 3d orbital of the MoS_2_ film. This shows the Mo 3d XPS spectrum of MoS_2_ located at 229 eV and 232.23 eV corresponding to the 3d_5/2_ and 3d_3/2_ electronic states of Mo in 2H-MoS_2_, respectively [56,58], which corresponded to Mo^4+^. In addition, the energy spectra of 232.74 eV and 235.82 eV correspond to the Mo 3d_5/2_ and Mo 3d_3/2_ of Mo^6+^, respectively [59]. This showed that MoS_x_O_y_ was contained in the MoS_2_ film of the sample [56]. It indicated that the surface chemical composition of sputtered MoS_2_ was susceptible to moisture and oxygen when stored in the atmosphere and may have chemically oxidized to MoS_x_O_y_. Moreover, there was a peak at 226.38 eV in the Mo 3d energy spectrum. The peak represented the S 2 s peak, and its appearance in the Mo 3d energy spectrum was due to the overlapping area of the two energies [60].

Figure 9d shows the S2p XPS spectra of two characteristic peaks. The binding energy was located at 162 eV and163.25 eV approximately, and it corresponded similarly to 2p_3/2_ and 2p_1/2_ of S, respectively. The spectra could be divided into four other peaks. Peaks at the binding energy = 162.0 eV and 163.25 eV corresponded to the Mo-S bond structure and the presence of the metallic 1 T phase of MoS_2_, whereas other doublet peaks at the binding energy = 163.28 eV and 164.58 eV corresponded to a stable MoS_2_ bond structure that arises from the semiconducting 2 H phase of MoS_2_ [56,61].

### 3.3. Piezoresistive Property

**Figure 10:**
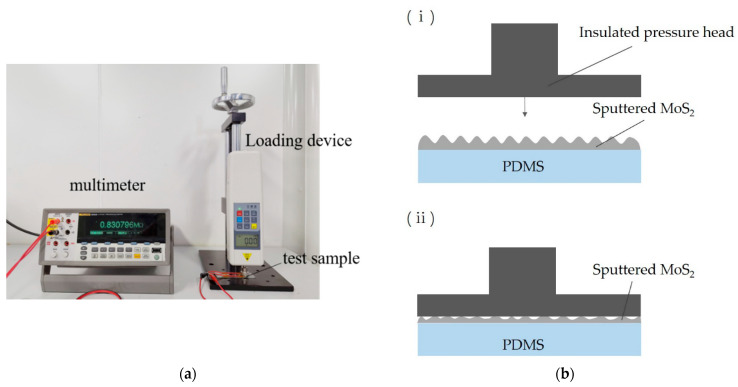

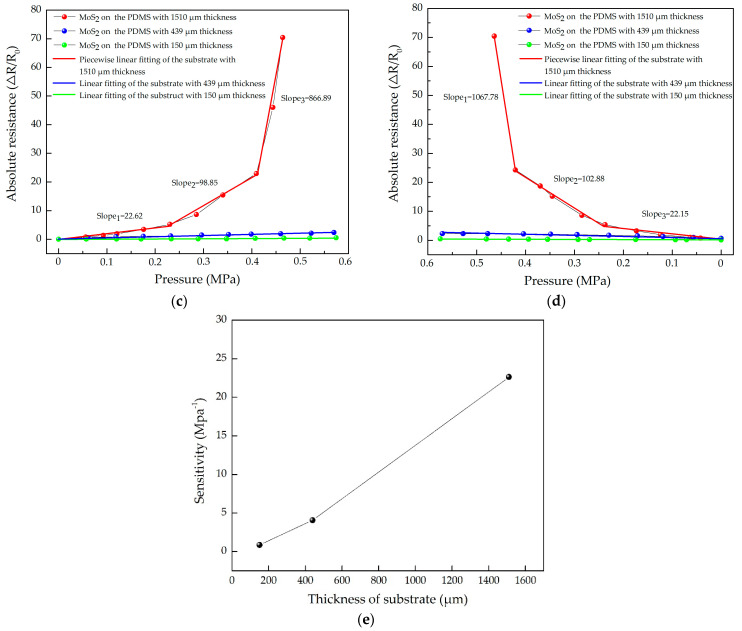
The piezoresistive property test of sputtered MoS2 film: (**a**) the piezoresistive testing system for thin films; (**b**) the schematic diagram of the film surface compression: (**i**) the state of Scheme 2 before pressure; (**ii**) the state of sputtered MoS2 under pressure; (**c**,**d**) pressure test curves of MoS2 films deposited on PDMS substrates with different thicknesses at the power of 350 W; (**e**) the relationship diagram between the thickness of the substrate and the sensitivity of MoS_2_ sputtered on the substrate at 0–0.23 MPa.

Figure 10a shows the piezoresistive performance test system of the flexible thin films. When the pressure *p* = 0, the MoS_2_ film attached to the PDMS substrate was not deformed, and the resistance relative change in the MoS_2_ film was 0. When the pressure acted on the surface of the film, as shown in Figure 10b, the deformation of the MoS_2_ film attached to the PDMS substrate occurred, resulting in a change in the resistance value of the MoS_2_ film resistance. The resistance R was measured by a multimeter, and we found that with the variation of pressure *p*, the resistance of the MoS_2_ film varied clearly. 

By calculating the resistances of the MoS_2_ film under different pressures, the relationship of the resistance relative changes ΔR/R, and the pressure *p* was obtained. With the substrate temperature at room temperature and the sputtering power was 350 W, variation curves of ΔR/R to the pressure of the MoS_2_ films on PDMS substrates with different thicknesses are shown in Figure 10c,d.

Figure 10c is a loading curve, while Figure 10d is an unloading curve. As can be seen from the figures, at the sputtering power of 350 W, the MoS_2_ film sputtered at room temperature on the thick PDMS substrate had a better piezoresistive performance. This is because when the MoS_2_ film was subjected to pressure variation, the MoS_2_ film on the thick substrate had greater strain, and its grain structure changed, leading to the change in the bandgap width. Then, the carrier transport mechanism of MoS_2_ was changed, which manifested as regular changes in the resistance of the MoS_2_ film.

Thicker films had better piezoresistive effects and higher sensitivity, the thin sensitivity (when the film was loaded) was small, and the value was about 0.86 MPa^−1^. In addition, the thickness of the film was divided into three sections; the pressure resistance coefficient below 0.23 MPa was 22.62 MPa^−1^. In the range of 0.23 to 0.4 MPa, the piezoresistive coefficient was 98.85 MPa^−1^; 0.4 MPa above it was 866.90 MPa^−1^. The piezoresistive coefficient of the MoS_2_ film on a thin substrate was small and almost linear. This is because molybdenum disulfide deposited on a PDMS substrate was strained with the flexible substrate under pressure, and the strain of a thinner flexible substrate was smaller.

For molybdenum disulfide thin films deposited on the thick flexible substrate, when the stress was small, the substrate strain was small, and so the film piezoresistive coefficient was small. When the stress increased, the strain of the flexible substrate increased, and the film piezoresistive coefficient increased. When the load on the film and the substrate continued to increase, the flexible substrate cracked, and the molybdenum disulfide thin film deposited on the substrate cracked; thereby, its piezoresistive coefficient suddenly increased, mainly due to the pressure. In addition, we obtained the relationship diagram between the thickness of the substrate and the sensitivity of MoS_2_ sputtered on the substrate at 0–0.23 MPa, as shown in Figure 10e. The sensitivity corresponding to the thickness of the substrate of 150, 439, and 1510 μm was 0.86, 4.05, and 22.62 MPa^−1^. We can see intuitively that the sensitivity of MoS_2_ sputtered on the substrate increased almost linearly with the increase in the substrate thickness. 

### 3.4. Pressure Sensor of MoS_2_ Film

**Figure 11:**
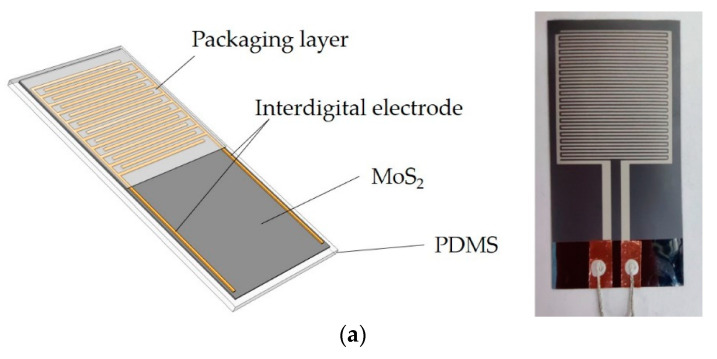

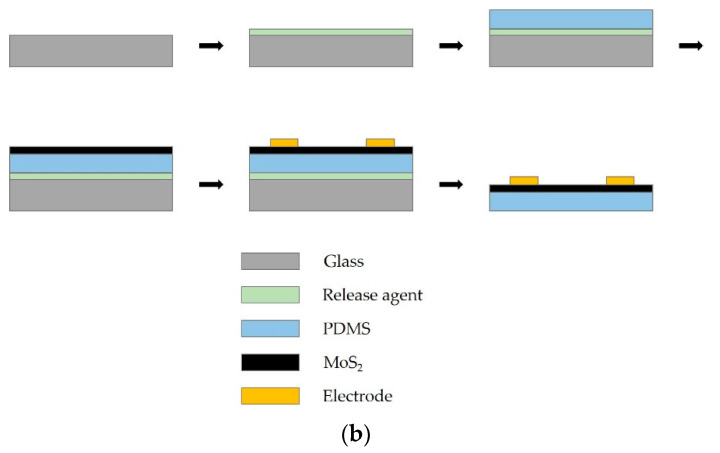
The schematic of the MoS_2_ film pressure sensor and its processing flow: (**a**) the MoS_2_ thin film pressure sensor; (**b**) Production process of MoS_2_ film pressure sensor.

We found that the MoS_2_ film sputtered on the natural coating PDMS flexible substrate (when the spinning speed was 0 r/min) had a significant piezoresistive effect. Based on this principle, we developed a sandwich-structured MoS_2_ film flexible piezoresistive sensor, as shown in Figure 11a. The sensor used PDMS as a flexible substrate, and the middle layer was the MoS_2_ film, which was the sensitive element. The upper layer was fabricated using screen-printing technology to form an interdigital electrode. Last, ultra-thin flexible PET film or PDMS insulating protective layer was covered on the upper layer, and the new type of high-sensitivity film-based dynamic pressure sensor was finished. Figure 11b shows the processing flow chart of the thin film sensor. A piece of glass was used as a rigid carrier, and the release agent formed an intermediate layer, which could separate the PDMS from the carrier easily. With sputtered MoS_2_ as a piezoresistive and sensitive structure, the thickness of the sensitive film was reduced to 200–1000 nm, and the thickness of the film sensor was reduced by an order of magnitude. Finally, the screen-printed interdigital electrodes had the advantages of simple structure, low cost, and can be well adapted to flexible sensor.

### 3.5. The Plantar Pressure Test of MoS_2_ Film Sensor

**Figure 12:**
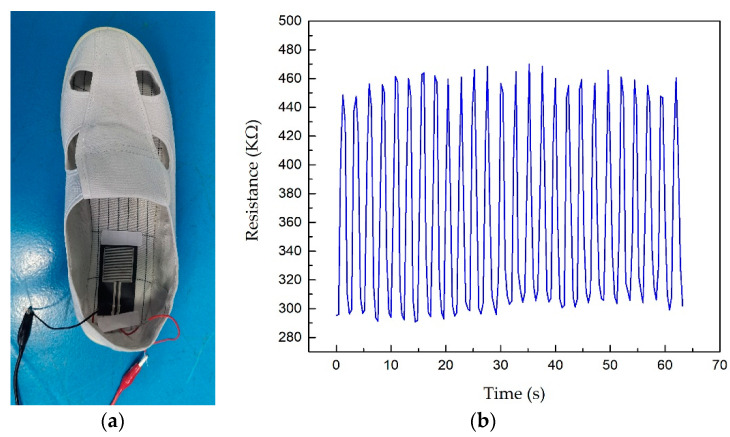
The plantar pressure test of the MoS_2_ film sensor. (**a**) The test system of plantar pressure; (**b**) output curve of the plantar pressure signal during walking.

From the piezoresistive performance test results of the MoS_2_ thin film deposited by magnetron sputtering, it can be seen that the MoS_2_ thin film pressure sensor had the advantages of low thickness, light weight, and good flexibility. Here, an MoS_2_ thin film pressure sensor was used to measure the plantar pressure of human soles. The thin film sensor was attached to the back position of a shoe, as shown in Figure 12a so that the plantar completely pressed the sensitive part of the sensor. Then, the continuous in-situ step action was performed to obtain the output signal of the sensor, as shown in Figure 12b. The MoS_2_ film pressure sensor had good signal output and high sensitivity. The plantar pressure situation could be obtained by the output signal of sensor and this will help the further processing and analysis of the signal and can obtain the relevant gait information. This is of great significance for gait analysis and rehabilitation medicine.

## 4. Conclusions

Here, an MoS_2_ film deposited by the magnetron sputtering method was used as a piezoresistive sensitive film for the first time. The MoS_2_ film was combined with a flexible PDMS substrate, and oxygen plasma treatment technology was used to improve the adhesion between PDMS and MoS_2_ effectively. A screen-printing method was used to make electrodes on the surface of the film to form a pressure sensor with a thickness of nanometers. The features of the MoS_2_ film were characterized SEM, Raman microscopy spectrometer, XRD, and XPS. 

With a sputtering power of 350 W, the morphology, structure, crystallinity, and chemical composition of the MoS_2_ thin films deposited on PDMS substrates were explored. An MoS_2_ ultra-thin film flexible pressure sensor was tested with the universal testing system. The MoS_2_ film demonstrated good piezoresistive performance when loading and unloading at 0–600 kPa. A flexible human pressure sensor based on an MoS_2_ film was designed and developed. The plantar pressure test showed that the sensor had good signal output and high sensitivity, which is of great significance for gait analysis and rehabilitation medicine.

## Figures and Tables

**Table 1:** Ratio of molybdenum (Mo) and sulfide (S) in different valence states in photoelectron spectroscopy (XPS) spectral data.

Name	BE (eV)	FWHM	Atomic (%)
Mo 3d5/2 (MoS_2_)	229.00	1.76	30.67
Mo 3d5/2 (MoSXOy)	232.74	1.29	6.00
S2p3/2 (MoS_2_)	162.00	1.26	54.53
S2p3/2 (MoSXOy)	163.28	0.96	8.80

BE—Binding Energy; FWHM—Full Width at Half Maximum.

## Data Availability

Not applicable.
